# Supplementary material for: Heterozygous loss of function of IQSEC2/Iqsec2 leads to increased activated Arf6 and severe neurocognitive seizure phenotype in females
Source: Life Sci Alliance. 2019 Aug 22;2(4):e201900386. doi: 10.26508/lsa.201900386 (PMC6706959; doi:10.26508/lsa.201900386)
Supplement: Supplementary file 7 [file LSA-2019-00386_Supplemental_Data_1.docx]

**Data file S1: Patient phenotype**

The proband (II-2), a 68-year-old female, was breech born as a 1.9kg birth weight twin at gestational age 34 weeks to a healthy 22-year-old mother and father with subnormal intelligence after an uncomplicated pregnancy. She learned to sit at 8-10 months and walk at age 18 months. She has a history of normal early development until seizure onset at 17 months. She then developed repeated, generalized seizures during the day-time and regression was observed. The proband later developed additional atypical absence and atonic seizure types. While sitting she showed peculiar hand movements. She had developed some words, which were lost after seizure onset. Thereafter, she never learned to speak and instead communicates using sounds and gestures. The diagnosis of severe and profound ID (SPID; IQ < 35) was made. Since infancy and early childhood her adaptive behaviour skills have remained profoundly limited in every indicator: sensorimotor development, communication skills, self-help skills, socialization, and interaction with others. The proband presents with motor hyperactivity, impulsive, intrusive, and self-stimulatory behaviours. She has a peculiar personality accompanied by some psychiatric disturbances, and in adolescent years this has been managed with antipsychotics. There is no indication of cognitive deterioration or progressive disability, but she displays some disruptive behaviour. Currently, the patient tends to scream inappropriately and eat inappropriate objects. She also bites her hands and picks her ears.

Since the age of 11, she has been living in residential care. Her daily functioning skills appear unchanged and poor. She needs assistance in every-day life. She eats smooth food, aided left-handed with a spoon, and drinks aided from a cup. Her right hand shows mild spasticity. She walks independently with a broad gait and has a history of frequent falling. In addition, she tends to lean to the right. She is not toilet-trained and needs aid in hygiene. Facial features include low-set, large ears, and asymmetric facial features with prominent angle of the jaw, thick upper lip with mild hypertrichosis over the upper lip and deep-set eyes (Figure 7B). No brain MRI has been performed due to the requirement of general anaesthesia.
